# Supplementary material for: P-TRAP: a Panicle Trait Phenotyping tool
Source: BMC Plant Biol. 2013 Aug 29;13:122. doi: 10.1186/1471-2229-13-122 (PMC3848748; doi:10.1186/1471-2229-13-122)
Supplement: Additional file 7 — P-TRAP source code. The Netbeans project that contains the source code of the application. [file 1471-2229-13-122-S7.zip › javadoc.zip!/javadoc/org-bioalg-imageprocessor/org/bioalg/imageprocessor/MathMorphology.html]

MathMorphology (Image Processor)


JavaScript is disabled on your browser.


- Package
- Class
- Tree
- Deprecated
- Index
- Help

*org.bioalg.imageprocessor 1.0*

- Prev Class
- Next Class

- Frames
- No Frames

- All Classes

- Summary:
- Nested |
- Field |
- Constr |
- Method

- Detail:
- Field |
- Constr |
- Method


org.bioalg.imageprocessor

## Class MathMorphology

- java.lang.Object
- - org.bioalg.imageprocessor.MathMorphology

- ---

    

  ```
  public class MathMorphology
  extends java.lang.Object
  ```

  The Mathematical morphology handler. It has all basic operations to perform
  the mathematical morphology. The structure element is modeled as a kernel see
  `Kernels`

  Author:
  :   faroq

- - ### Nested Class Summary

    Nested Classes

    | Modifier and Type | Class and Description |
    | --- | --- |
    | `static class` | `MathMorphology.MorphingType` |
  - ### Constructor Summary

    Constructors

    Constructor and Description

    `MathMorphology()`

    `MathMorphology(AImage sourceAImage)`
  - ### Method Summary

    Methods

    | Modifier and Type | Method and Description |
    | --- | --- |
    | `AImage` | `close(int[][] kernel)` The closing task. |
    | `AImage` | `dilate(int[][] kernel)` Dilate a binary image by using a structure element. |
    | `AImage` | `erode(int[][] kernel)` Eroding the image by a structure element. |
    | `AImage` | `erode1(int[][] kernel)` to be reworked |
    | `AImage` | `getSourceAImage()` |
    | `AImage` | `open(int[][] kernel)` The Opening task. |
    | `AImage` | `outline(int[][] kernel)` Calculate the contour of binary particles by using simple math morphology tasks. |
    | `void` | `setSourceAImage(AImage sourceAImage)` |

    - ### Methods inherited from class java.lang.Object

      `clone, equals, finalize, getClass, hashCode, notify, notifyAll, toString, wait, wait, wait`

- - ### Constructor Detail


    - #### MathMorphology

      ```
      public MathMorphology()
      ```


    - #### MathMorphology

      ```
      public MathMorphology(AImage sourceAImage)
      ```
  - ### Method Detail


    - #### getSourceAImage

      ```
      public AImage getSourceAImage()
      ```


    - #### setSourceAImage

      ```
      public void setSourceAImage(AImage sourceAImage)
      ```


    - #### dilate

      ```
      public AImage dilate(int[][] kernel)
      ```

      Dilate a binary image by using a structure element.

      Parameters:
      :   `kernel` - the structure element.

      Returns:
      :   a dilated version of the image.


    - #### erode1

      ```
      public AImage erode1(int[][] kernel)
      ```

      to be reworked

      Parameters:
      :   `kernel` -

      Returns:


    - #### erode

      ```
      public AImage erode(int[][] kernel)
      ```

      Eroding the image by a structure element.

      Parameters:
      :   `kernel` - the structure element

      Returns:
      :   an eroded version of the input image.


    - #### open

      ```
      public AImage open(int[][] kernel)
      ```

      The Opening task.

      Parameters:
      :   `kernel` - the structure element

      Returns:
      :   an open image.


    - #### close

      ```
      public AImage close(int[][] kernel)
      ```

      The closing task.

      Parameters:
      :   `kernel` - the structure element

      Returns:
      :   a closed version


    - #### outline

      ```
      public AImage outline(int[][] kernel)
      ```

      Calculate the contour of binary particles by using simple math morphology
      tasks.

      Parameters:
      :   `kernel` -

      Returns:


- Package
- Class
- Tree
- Deprecated
- Index
- Help

*org.bioalg.imageprocessor 1.0*

- Prev Class
- Next Class

- Frames
- No Frames

- All Classes

- Summary:
- Nested |
- Field |
- Constr |
- Method

- Detail:
- Field |
- Constr |
- Method
